# Impact of Use of Gastric-Acid Suppressants and Oral Anti-Cancer Agents on Survival Outcomes: A Systematic Review and Meta-Analysis

**DOI:** 10.3390/cancers12040998

**Published:** 2020-04-18

**Authors:** Alice Indini, Fausto Petrelli, Gianluca Tomasello, Erika Rijavec, Antonio Facciorusso, Francesco Grossi, Michele Ghidini

**Affiliations:** 1 Oncology Unit, Fondazione IRCCS Ca’ Granda Ospedale Maggiore Policlinico, 20122 Milan, Italy; alice.indini@gmail.com (A.I.); erika.rijavec@policlinico.mi.it (E.R.); francesco.grossi@policlinico.mi.it (F.G.); 2 Oncology Unit, ASST Bergamo Ovest, 24047 Treviglio (BG), Italy; faupe@libero.it; 3 Oncology Unit, Niguarda Cancer Center, Grande Ospedale Metropolitano Niguarda, 20121 Milan, Italy; gianluca.tomasello@ospedaleniguarda.it; 4 Gastroenterology Unit, Università Degli Studi di Foggia, 71122 Foggia, Italy; antonio.facciorusso@virgilio.it

**Keywords:** gastric acid suppressant, chemotherapy, tyrosine kinase inhibitors, proton pump inhibitors

## Abstract

We performed a systematic review and meta-analysis to evaluate the role of gastric acid suppressant use on outcomes of tyrosine kinase inhibitors (TKIs) and oral chemotherapy. We identified all research evaluating the effect of GAS (gastric acid suppressants) use on patients receiving oral chemotherapy or TKIs for solid tumors. The pooled hazard ratios (HRs) and 95% confidence interval (95%CI) for overall survival (OS) and progression-free survival (PFS) were calculated with a fixed-effects or a random effects model. The study population included *n* = 16 retrospective studies and 372,418 patients. The series concerned gastrointestinal tract tumors (*n* = 5 studies), renal cell carcinomas (RCC, *n* = 3 studies), non-small cell lung cancers (NSCLC, *n* = 5 studies), and soft tissue sarcomas or mixed histologies solid tumors in *n* = 3 studies. The pooled HRs for OS and PFS were 1.31 (95%CI: 1.20–1.43; *p* < 0.01) and 1.3 (95%CI 1.07–1.57; *p* < 0.01) for GAS and no GAS users, respectively. Only studies of EGFR (epidermal growth factor receptor) mutated NSCLC patients receiving TKIs and those with colorectal cancer receiving oral chemotherapy showed a significant correlation between GAS and poor survival. Our study supports the evidence of a possible negative impact of concomitant GAS therapy on survival outcomes of patients receiving oral anti-cancer drugs.

## 1. Introduction

Oral chemotherapy has historically been part of therapeutic regimens for the treatment of cancer [1,2,3]. Over the last years, new oral anti-cancer agents acting as multi-tyrosine kinase inhibitors (TKIs) have dramatically changed patient prognosis and thereby have become standard treatments for several types of tumors [4,5,6,7,8,9]. TKIs targeting the epidermal growth factor receptor (EGFR) (e.g., gefitinib, erlotinib, afatinib, osimertinib) are currently approved for treatment of EGFR mutant non-small cell lung carcinoma (NSCLC), and multi-targeted TKIs (e.g., sunitinib, axitinib, sorafenib, pazopanib) for the treatment of renal cell carcinoma (RCC). Moreover, several new TKIs are currently being tested in clinical trials in several types of solid tumors. The use of oral drugs has a positive impact on patient quality of life for the convenience of self-administration; however, there is a significant risk of drug–drug interactions. The diffusion of these drugs often parallels that of gastric acid suppressants (GAS), such as proton pump inhibitors (PPI) or histamine-2 receptor antagonists (H2RA). GAS commonly represent part of the complex drug regimen of an average oncologic patient, with an estimated rate of 50% inappropriate PPIs prescriptions, both in hospital and ambulatory settings [10]. Because of the oral administration and pH-dependent solubility of chemotherapy and TKIs, concerns have been raised over the possible effect of co-administering drugs which raise gastric pH] [11,12]. Chronic acid suppression can reduce the effectiveness of drugs that require an acidic pH for their absorption [13]. Retrospective data suggest that TKI plasma concentration is decreased in patients receiving concomitant GAS therapy with subsequently poorer oncologic outcomes [14,15], however pooled analyses of patients enrolled in clinical trials have shown inconsistent results [16,17].

The aim of our meta-analysis is to define whether concomitant use of GAS therapy (either PPI or H2RA) in patients receiving treatment with oral anti-cancer agents (i.e., chemotherapy or TKIs) is associated with survival outcomes.

## 2. Results

**Figure 1:**
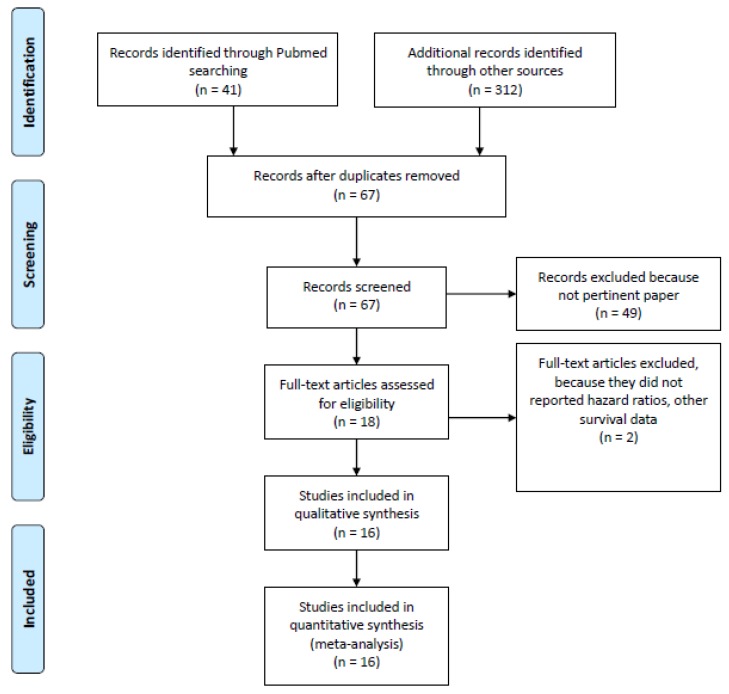
Flow diagram of included studies.

A total of 353 potentially eligible records were identified in the electronic databases. After exclusion of *n* = 337 not pertinent papers, *n* = 16 were selected for inclusion in quantitative analysis (*n* = 372,418 patients included, with 12% of patients receiving concomitant GAS therapy) [16,17,18,19,20,21,22,23,24,25,26,27,28,29,30,31]. The search results and characteristics of the included studies are presented in Figure 1 and Table 1 and Table 2.

All studies were retrospective except for a pooled analysis of phase 2–3 studies by Lalani et al. [16] and a secondary analysis of a randomized prospective trial by Chu et al. [19]. Oncologic treatment consisted of oral TKIs in *n* = 11 studies, while in *n* = 4 studies patients received oral chemotherapy (i.e., capecitabine); one study did not include information regarding the type of study drugs. Oncologic diagnoses were cancers of the gastrointestinal tract (GI, *n* = 5 studies), RCC (*n* = 3 studies), NSCLC (*n* = 5 studies), and soft tissue sarcomas or mixed histologies solid tumors in *n* = 3 studies. Quality according to NOS scale was moderate (range 5–8; median 6).

### 2.1. Overall Survival and Progression-Free Survival with GAS vs. no GAS

**Figure 2:**
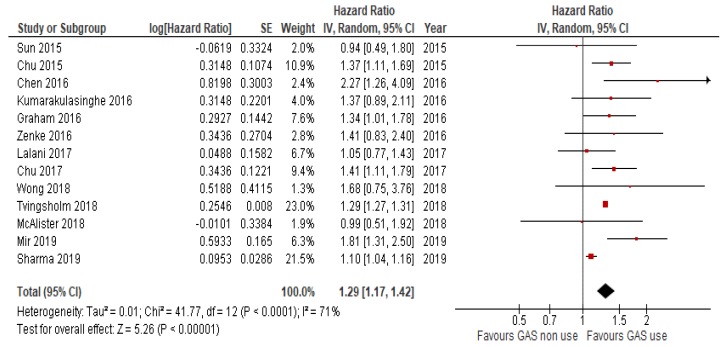
Forest plot for overall survival of the analyzed studies.

**Figure 3:**
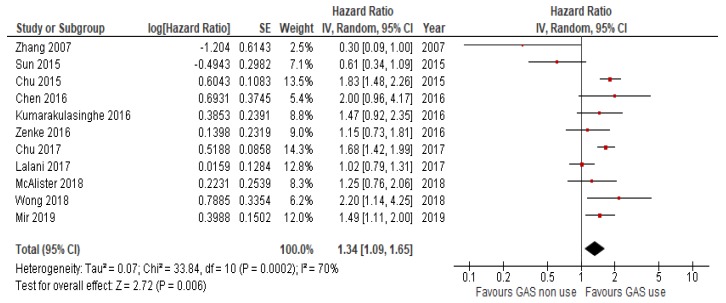
Forest plot for progression free survival of the analyzed studies.

*N* = 15 studies reported data on OS. Because the heterogeneity test showed a high level of heterogeneity (I^2^ = 68%, *p* < 0.01) among studies, a random effects model was used for the analysis. The OS of patients receiving concomitant GAS therapy was significantly worse (HR = 1.31, 95%CI: 1.20–1.43; *p* < 0.01; Figure 2) compared to those of patients not receiving GAS. Similarly, the use of GAS reduced PFS in *n* = 13 studies that reported data on PFS (HR = 1.3, 95%CI 1.07–1.57; *p* < 0.007; Figure 3). Heterogeneity was high (I^2^ = 74%), so a random effects model was used. 

### 2.2. Subgroup Analysis

In a separate analysis of studies involving patients treated with TKIs, the use of concomitant GAS was similarly associated with poorer OS (HR = 1.35, 95%CI 1.16–1.56; *p* < 0.01). Similarly, capecitabine assumption with GAS resulted in increased mortality (HR = 1.37, 95%CI 1.1–1.7; *p* < 0.01). We also searched for a distinct correlation of concomitant GAS in different tumor types: only studies of EGFR-mutated NSCLC patients receiving TKIs and either PPIs or H2RAs and those with GI cancers receiving all PPIs and oral chemotherapy retained a significant correlation between GAS and poor survival (HR = 1.47, 95%CI 1.27–1.71; *p* < 0.01 and HR = 1.3, 95%CI 1.02–1.66; *p* = 0.04), while in the case of renal cell carcinoma, the correlation between GAS assumption and reduced survival was missing. In patients with lung cancer on anti-EGFR, regression between H2RA and HR for OS was not significant, so the contribution of H2RA does not seem relevant for the final outcome. 

In some studies, both PPIs and H2RAs were administered. After exclusion of these studies, *n* = 7 publications included only patients taking PPIs, and HR for OS was similar to the whole population (HR = 1.22, 95%CI 1.09–1.36; *p* < 0.01). In studies that reported median follow-up (*n* = 6), OS was still poorer in patients taking GAS (HR = 1.29, 95%CI 1.27–1.31; *p* < 0.01). 

### 2.3. Overall Response Rate

**Figure 4:**
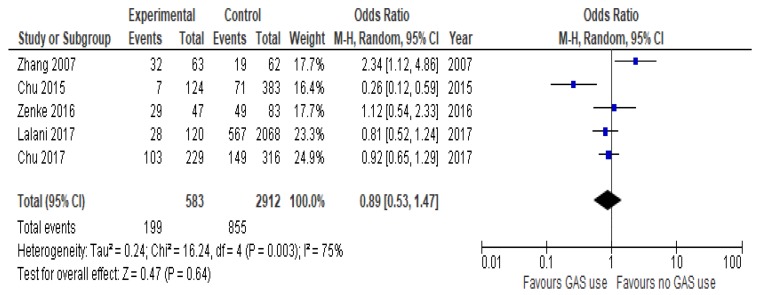
Forest plot for overall response rate of the analyzed studies.

In few studies with data available, PPIs did not influence ORR (OR = 0.89, 95%CI 0.53–1.47; *p* = 0.64, Figure 4). 

### 2.4. Publication Bias

**Figure 5:**
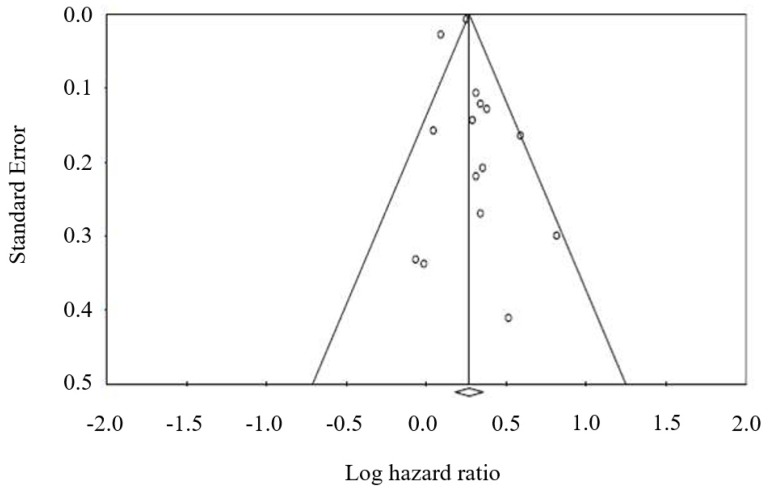
Funnel plot for publication bias in overall survival analysis.

A funnel plot was used to assess publication bias in the studies evaluating OS with concomitant GAS versus no GAS therapy in cancer patients. No publication bias was detected. Furthermore, Egger’s test was not significant (*p* = 0.39) (Figure 5). 

## 3. Discussion

This is the first meta-analysis exploring the role of concomitant GAS therapy during administration of oral anti-cancer agents for treatment of solid tumors. According to our results, GAS therapy seems to negatively impact on OS and PFS, while it has no impact on ORR. 

GAS, and above all PPIs, are among the most commonly prescribed drugs worldwide. Their principal application is treatment of gastroesophageal inflammatory syndromes, such as gastroesophageal reflux disease, esophagitis, and peptic ulcer disease [32]. Given their mild toxicity profile, the use of PPIs has spread over the last 20 years, and we are now facing an overuse in patients with benign conditions or who do not need this specific therapy. Recently, various studies have related PPI use to increased incidence of respiratory tract and *Clostridium difficile* infections, mainly related to an altered commensal intestinal microbiome, as a consequence of raised gastric pH and bacterial overgrowth [33]. 

The clinical impact of concomitant use of GAS therapy and oral anti-cancer agents remains controversial. Numerous pharmacokinetic studies have addressed this question, showing a possible detrimental effect of GAS on oral anti-cancer drug absorption. However, this phenomenon varies according not only to the drugs analyzed, but depends also on specific drug–drug interactions differing among drugs of the same class [11,12,34,35]. As an example, Egorin et al. showed that PPIs may significantly decrease dasatinib plasmatic levels, while they do not impact on imatinib levels [34]. A similar effect was shown in a small series of patients using concomitant GAS and erlotinib [11], but was not confirmed by data of patients included in the BR.21 trial database [35]. This retrospective analysis on clinical outcomes of patients receiving concomitant GAS and erlotinib showed no differences in plasma drug levels and survival outcomes compared with patients who did not take concomitant GAS [35]. However, the pH-dependent absorption of erlotinib was confirmed in a randomized pharmacokinetic study, which demonstrated that concomitant Cola intake led to a clinically relevant increase in erlotinib bioavailability during esomeprazole treatment due to a temporarily lowered intragastric pH [36]. Analyses on the pharmacokinetics of different TKIs showed that afatinib is highly soluble throughout the physiologic pH range and may therefore have fewer interactions with GAS, compared with gefitinib or erlotinib [37]. A similar effect was observed for osimertinib, where plasmatic levels were not determined by food or PPI co-administration [12]. With our meta-analysis, we reported a significant correlation between GAS and poor survival only for the NSCLC and CRC subgroups, while there was no significant impact on survival when RCC series were considered. A possible explanation may be found in the difference between oral TKIs used in NSCLCs and RCCs. Indeed, TKIs used in lung cancer own anti-EGFR activity (gefitinib and erlotinib), while TKIs used in RCCs have mainly anti-vascular endothelial growth factor (VEGF) properties (sunitinib, sorafenib, axitinib and pazopanib). Moreover, our results are consistent with findings of a previous pooled analysis of metastatic RCC patients treated in phase II and III trials. Indeed, OS results were similar between PPI and non-PPI users in the case of anti-VEGF TKI use [16].

There are two main concerns related to alterations in pharmacokinetics during concomitant GAS therapy. The first is that combined use of PPIs and TKIs may increase the treatment-related adverse events (AEs) of both drugs. Although intuitive, this mechanism is also controversial: in a recent report from Cho et al., concomitant GAS therapy increased gefitinib-induced hepatotoxicity [38]. However, another case series of patients treated with gefitinib and erlotinib did not show differences in the incidence of cutaneous AEs and diarrhea, when comparing patients receiving concomitant GAS to those who did not [30]. Similar reports of patients undergoing concomitant capecitabine and PPIs showed that rates of treatment discontinuation and/or dose reduction due to toxicities were comparable to that of patients not receiving GAS therapy [20,29].

The second important issue lies in the potentially reduced absorption and subsequent compromised anti-cancer drug effect. Reports from the literature on this topic mainly consist of case series, reporting heterogeneous data in terms of patient populations, anti-cancer drugs (chemotherapy, TKIs), GAS therapy (PPIs, H2RA, or both), and outcomes (survival vs response vs AEs incidence). Our meta-analysis confirmed that concomitant GAS can have a negative impact on PFS and OS, however without significant effects on ORR. One of the possible reasons for the worse survival outcomes is that patients requiring GAS are older and have various comorbidities (e.g., cardiovascular disease requiring aspirin and therefore PPI therapy). Another theory is that concomitant GAS therapy reduces serum levels of anti-cancer drugs under the therapeutic threshold, thus increasing the risk for distant metastasis and disease progression. Although previous studies show that TKIs are effective even at low serum levels, it is recognized that the cerebrospinal fluid penetration rate of first-generation TKIs is only around 2% [39]. Thus, the concomitant use of drugs reducing gastric absorption of TKIs may further reduce their serum levels to an insufficient plasmatic concentration [40]. 

Given these two considerations, we can speculate that concomitant administration of GAS drugs during anti-cancer therapy does not significantly affect ORR because of the maintenance of an adequate therapeutic anti-cancer threshold. On the contrary, over a long period, GAS administration might affect therapeutic activity of anti-cancer drugs. This element, combined with risk factors of age and comorbidities of patients treated with GAS, might explain the worsened survival rates for this subgroup of patients. The relatively low number of studies reporting complete OS results (6 out of 16 analyzed studies), however, makes the interpretation of this result even more speculative. The observations of our analysis are only hypotheses-generating: data available so far can be used as starting points to carry on further prospective parallel data collection and analyses in clinical practice.

Our meta-analysis has some intrinsic limitations. First of all, patients taking PPIs may have an intrinsically poor performance status and/or chronic conditions that require continuous GAS. Secondly, use of PPIs was not offered with a randomized design so that patients treated with PPIs may have suffered from concomitant gastritis/dyspepsia and/or may have taken steroids for supportive care, consequently needing chronic GAS therapy. Thirdly, there is uncertainty regarding the correct administration of PPIs right before antitumoral treatment. 

Moreover, PPIs and H2RAs have different mechanisms of action and potency. Due to the heterogeneity of studies analyzed, we only have the results of a subgroup analysis of studies analyzing the effect of single GAS therapy (i.e., PPIs or H2RAs), with only limited data on length of overlapping therapies.

Finally, other pharmacological interactions (e.g., with the CYP3A4 citocrome) may have reduced plasmatic concentration of anti-EGFR agents.

## 4. Materials and Methods 

This study followed the Meta-analysis Of Observational Studies in Epidemiology (MOOSE) group guidelines and checklist [41] (Figure 1, Table 3).

### 4.1. Data Extraction and Quality Assessment

A protocol was defined prior to the search including the population criteria, description of oncologic treatments, comparisons, and outcomes of interest. A systematic literature search was performed using PubMed (MEDLINE), EMBASE and The Cochrane Library. The search was performed comprehensively using several databases from each one’s earliest start until 1st August 2019. We sought to identify all English language research evaluating the effect of GAS use on the outcomes of patients receiving concomitant oral chemotherapy or TKIs for solid tumors. For the process of evidence acquisition, the literature was queried using the following terms [MeSH]: “gastric acid suppressant” OR “proton pump inhibitors”, and “chemotherapy” or “tyrosine kinase inhibitors” AND “carcinoma” or “cancer” AND “survival”. References of included studies were hand-searched in order to identify potentially relevant adjunctive papers. For each study we extracted the following information, if available: number of patients, baseline patient characteristics, data regarding oncologic treatments, progression-free (PFS) or recurrence-free (RFS) survivals and overall survival (OS) or the corresponding HRs, and overall response rates (ORRs) in the 2 arms. 

Two independent reviewers (AI and FP) evaluated all studies in order to verify the inclusion criteria. Study selection was conducted with a two-phase screening. First level screening excluded titles and abstracts meeting the following criteria: (a) case reports, letters, comments, and reviews not reporting original data; (b) in vivo and/or in vitro studies; (c) studies involving fewer than 10 patients; and (d) language publication other than English. Studies matching inclusion criteria were obtained in the complete form and reviewed in their full-text version for an advanced assessment. Second level full-text screening was performed in order to include studies with the following criteria: (1) studies involving patients with solid tumors receiving oral chemotherapy or TKIs; (2) studies reporting outcomes of patients receiving concomitant GAS therapy compared to those who did not; (3) information regarding HRs or survival curves for OS and/or PFS and/or ORRs for patients using GAS compared to those who did not. Differences of opinion were resolved by agreement between the reviewers. Study quality was independently evaluated using the Newcastle-Ottawa Quality Assessment scale for case-control studies [42]. Disagreement was also resolved by consultation and consensus.

### 4.2. Statistical Analysis

The primary outcome of interest was OS. The secondary endpoints were PFS and ORR. The HRs and 95% CIs from each study were either extracted directly from original papers or calculated using Kaplan–Meier curves based on the method of Tierney et al [43]. Random effects models with inverse variance weighting were calculated using Review Manager (RevMan 5.3, The Nordic Cochrane Center, Copenhagen, Denmark). The heterogeneity of the underlying population was assessed using the Q-statistic and I^2^ test. For the interpretation, I^2^ values greater than 50% were considered to be heterogeneous [44]. Publication bias was assessed by visually evaluating a funnel plot (Begg’s and Egger’s test, Figure 4).

## 5. Conclusions

The use of GAS during cancer therapy with capecitabine or TKIs should be offered with caution because it may result in a reduction of anti-cancer treatment and may significantly affect therapeutic outcomes. In our meta-analysis, we observed a significantly worse OS and PFS in patients receiving GAS during cancer treatment with anti-EGFR TKIs or capecitabine-based regimens in GI cancers and NSCLC. In conclusion, except for clear clinical reasons (concomitant use of steroids/non-steroidal anti-inflammatory drugs, severe gastroesophageal reflux disease/gastritis/peptic ulcer) GAS should be avoided during treatment with oral anti-cancer drugs for solid tumors.

## Figures and Tables

**Table 1:** Main characteristics of the included studies.

Author	Principal Institution(s) Involved	Study Design	Study Period	Number of Patients	Patients’ Disease Characteristics	Oral Anti-cancer Drug	Type of GAS
Ha, 2014 [23]	Cross Cancer Institute, Department of Oncology, Edmonton, Alberta, Canada	retrospective	2006–2013	383	mRCC	Sunitinib	PPI
Sun, 2016 [27]	Cross Cancer Institute, Department of Oncology, Edmonton, Alberta, Canada	retrospective	2008–2012	298	Early stage CRC	Capecitabine	PPI
Chu, 2015 [19]	Cross Cancer Institute, Department of Oncology, Edmonton, Alberta, Canada	retrospective	2007–2012	507	EGFR mutant advanced NSCLC	Erlotinib	PPI, H2RA
Zenke, 2016 [30]	Department of Thoracic Oncology, National Cancer Center Hospital East, Kashiwa, Japan	retrospective	2008–2011	130	EGFR mutant advanced NSCLC	Gefitinib Erlotinib	PPI, H2RA
Kumarakulasinghe, 2016 [24]	Department of Haematology-Oncology, National University Cancer Institute, Singapore	retrospective	2008–2013	157	EGFR mutant advanced NSCLC	Gefitinib Erlotinib	PPI, H2RA
Chen, 2016 [18]	Chang Gung Memorial Hospital-Kaohsiung Medical Center, Chang Gung University College of Medicine, Kaohsiung, Taiwan	retrospective	2010–2013	269	EGFR mutant advanced NSCLC	EGFR TKIs NOS	PPI
Graham, 2016 [21]	Department of Oncology, Cancer Centre of Southeastern Ontario, Queen’s University, Kingston	retrospective	2005–2011	117	CRC	NA	PPI
Chu, 2017 [20]	Cross Cancer Institute, Department of Oncology, Edmonton, Alberta, Canada	retrospective analysis (phase III trial)	2008–2012	545	GEJC	Capecitabine	PPI
Zhang, 2017 [31]	Guangdong Medical University Affiliated Longhua Central Hospital, Shenzhen, China	retrospective	2008–2016	125	CRC	Capecitabine	PPI
Lalani, 2017 [16]	Department of Medical Oncology, Dana-Farber Cancer Institute, Boston, USA	pooled analysis (phase II/III studies)	2003–2013	2188	mRCC	Sunitinib Axitinib Sorafenib	PPI
McAlister, 2018 [25]	Vanderbilt-Ingram Cancer Center, Nashville, USA	retrospective	2010–2015	90	mRCC	Pazopanib	PPI, H2RA
Tvingsholm, 2018 [28]	Danish Cancer Society Research Center, Copenhagen, Denmark (Danish Cancer Registry)	retrospective	1995–2011	353,071	Solid Tumors (Danish Cancer Registry)	NA	PPI
Wong, 2019 [29]	Cross Cancer Institute, Department of Oncology, Edmonton, Alberta, Canada	retrospective	2004–2013	389	stage II-III CRC	Capecitabine	PPI
Fang, 2019 [21]	Chang Gung Memorial Hospital, Chiayi Branch, Puzi City, Chiayi County, Taiwan	retrospective	1997–2013	1278	EGFR mutant advanced NSCLC	Gefitinib	PPI
Mir, 2019 [17]	Gustave Roussy, Sarcoma Group, Villejuif, France	retrospective	2005–2007 2008–2010	333	STS	Pazopanib	PPI, H2RA
Sharma, 2019 [26]	The University of Mississippi, Oxford, Mississippi, USA (SEER Database)	retrospective	2007–2012	12,538	Solid Tumors (SEER Database)	TKIs	PPI

Legend: CRC, colorectal cancer; GEJC, gastro-esophageal junction cancer; EGFR, epidermal growth factor receptor; GAS, gastric acid suppressants; H2RA, histamine-2 receptor antagonists; NA, not applicable; NOS, not otherwise specified; NSCLC, non-small cell lung cancer; PPI, proton-pump inhibitors; mRCC, metastatic renal cell carcinoma; SEER, Surveillance, Epidemiology, and End Results; STS, soft-tissue sarcoma; TKI, tyrosine kinase inhibitors; USA, United States of America.

**Table 2:** Response and survival outcomes in the analyzed studies.

Authors, Year	Median Follow-Up, Months	Criteria for Overlapping between GAS and Anti-cancer Treatment (Time Overlapping %)	Therapeutic Approach, *n* (%)	ORR	OS HR (95% CI) *	PFS HR (95% CI) *	Type of Analysis	Quality NOS Score
Ha, 2014 [23]	NA		GAS: 45 (20%)	NA	1.43 (0.95–2.15)	1.36 (0.92–2.01)	UVA	5
		100	No GAS: 186 (80%)	NA				
Sun, 2016 [27]	NA		GAS: 77 (26%)	NA	0.94 (0.49–1.78)	0.61 (0.34–1.08)	MVA	5
		Any PPI prescription	No GAS: 202 (74%)	NA				
Chu, 2015 [19]	NA		GAS: 124 (25%)	5.6%	1.37 (1.11–1.69)	1.83 (1.48–2.25)	MVA	6
		≥20	No GAS: 383 (75%)	18.5%				
Zenke, 2016 [30]	36 (10.1–85.2)		GAS: 47 (36%)	64%	1.41 (0.83–2.35)	1.15 (0.73–1.79)	MVA	7
		PPI/H2RA sequentially or concurrently to anti-EGFR	No GAS: 83 (64%)	63%				
Kumarakulasinghe, 2016 [24]	50		GAS: 55 (35%)	NA	1.37 (0.89–2.12)	1.47 (0.92–2.35)	MVA	7
		≥30	No GAS: 102 (65%)	NA				
Chen, 2016 [18]	24.5		GAS: 57 (21%)	NA	2.27 (1.26–4.11)	2.00 (0.96–4.17)	MVA	6
		≥30	No GAS: 212 (79%)	NA				
Graham, 2016 [21]	NA		GAS: 117 (9%)	NA	1.34 (1.01–1.79)	NA	MVA	7
		NA	No GAS: 1187 (91%)	NA				
Chu, 2017 [20]	NA		GAS: 119 (44%)	36%	1.41 (1.11–1.71)	1.68 (1.42–1.94)	MVA	5
		≥20	No GAS: 155 (56%)	42%				
Zhang, 2017 [31]	66		GAS: 29 (23%)	52.2%	0.30 (0.09–0.99)	0.37 (0.11–1.23) *	UVA *, MVA	7
		≥200 mg PPI	No GAS: 96 (77%)	36.5%				
Lalani, 2017 [16]	NA		GAS: 120 (5%)	23.3%	1.05 (0.77–1.44)	1.02 (0.79–1.30)	MVA	5
		≥1 dose PPI	No GAS: 2068(95%)	27.4%				
McAlister, 2018 [25]	NA		GAS: 66 (73%)	NA	0.99 (0.51–1.93)	1.25 (0.76–2.07)	MVA	5
		≥90 days	No GAS: 24 (27%)	NA				
Tvingsholm, 2018 [28]	1.52 (0.50–3.89)		GAS: 41,218 (11.7%)	NA	1.29 (1.27–1.31)	NA	MVA	7
		≥2 prescriptions within 6 months	No GAS: 311,853 (88.3%)	NA				
Wong, 2019 [29]	NA		GAS: 50 (23.4%)	NA	1.68 (0.75–3.80)	2.20 (1.14–4.25)	MVA	5
		Any time PPI during capecitabine	No GAS: 164 (76.6%)	NA				
Fang, 2019 [21]	NA		GAS: 309 (24%)	NA	1.67 (1.33–2.09)	0.99 (0.80–1.23)	MVA	7
		≥20	No GAS: 969 (76%)	NA				
Mir, 2019 [17]	27.6 (22.9–35.4)		GAS: 59 (18%)	NA	1.81 (1.31–2.49)	1.49 (1.11–1.99)	MVA	6
		≥80	No GAS: 273 (82%)	NA				
Sharma, 2019 [26]	NA		GAS: 2843 (22.7%)	NA	1.10 (1.04–1.17)	NA	MVA	8
		≥30 days within 3 months	No GAS: 9695 (77.3%)	NA				

* When both univariate and multivariate analyses were performed, HR results of multivariate analyses are reported. Legend: CI, confidence interval; GAS, gastric acid suppressants; HR, hazard ratio; NA, not available; NA, not determined; NOS, Newcastle-Ottawa Scale; MVA, multivariate analysis; ORR, overall response rate; OS, overall survival; PFS, progression free survival; UVA, univariate analysis.

**Table 3:** MOOSE Checklist for Meta-analyses of Observational Studies.

Item No	Recommendation	Reported on Page No
Reporting of background should include		
1	Problem definition	1,2
2	Hypothesis statement	1,2
3	Description of study outcome(s)	11
4	Type of exposure or intervention used	11
5	Type of study designs used	11
6	Study population	11
Reporting of search strategy should include		
7	Qualifications of searchers (e.g., librarians and investigators)	1
8	Search strategy, including time period included in the synthesis and key words	11
9	Effort to include all available studies, including contact with authors	11
10	Databases and registries searched	11
11	Search software used, name and version, including special features used (e.g., explosion)	11
12	Use of hand searching (e.g., reference lists of obtained articles)	11, Figure 1
13	List of citations located and those excluded, including justification	11, Figure 1
14	Method of addressing articles published in languages other than English	11
15	Method of handling abstracts and unpublished studies	11
16	Description of any contact with authors	11
Reporting of methods should include		
17	Description of relevance or appropriateness of studies assembled for assessing the hypothesis to be tested	11
18	Rationale for the selection and coding of data (e.g., sound clinical principles or convenience)	11
19	Documentation of how data were classified and coded (e.g., multiple raters, blinding and interrater reliability)	11
20	Assessment of confounding (e.g., comparability of cases and controls in studies where appropriate)	11
21	Assessment of study quality, including blinding of quality assessors, stratification or regression on possible predictors of study results	11
22	Assessment of heterogeneity	7,8, Figure 5
23	Description of statistical methods (e.g., complete description of fixed or random effects models, justification of whether the chosen models account for predictors of study results, dose-response models, or cumulative meta-analysis) in sufficient detail to be replicated	12
24	Provision of appropriate tables and graphics	Figure 1
Reporting of results should include		
25	Graphic summarizing individual study estimates and overall estimate	Table 1 and Table 2
26	Table giving descriptive information for each study included	Table 1 and Table 2
27	Results of sensitivity testing (e.g., subgroup analysis)	2, 6–8, Figure 2, Figure 3 and Figure 4
28	Indication of statistical uncertainty of findings	7,8, Figure 5
29	Quantitative assessment of bias (e.g., publication bias)	7,8, Figure 5
30	Justification for exclusion (e.g., exclusion of non-English language citations)	Figure 1, 11
31	Assessment of quality of included studies	11
Reporting of conclusions should include		
32	Consideration of alternative explanations for observed results	8,9
33	Generalization of the conclusions (i.e., appropriate for the data presented and within the domain of the literature review)	12
34	Guidelines for future research	8,9,11
35	Disclosure of funding source	12
